# The Role of the Gut Microbiota in Sanfilippo Syndrome’s Physiopathology: An Approach in Two Affected Siblings

**DOI:** 10.3390/ijms25168856

**Published:** 2024-08-14

**Authors:** Raquel Barbero-Herranz, María Garriga-García, Ana Moreno-Blanco, Esther Palacios, Pedro Ruiz-Sala, Saioa Vicente-Santamaría, Sinziana Stanescu, Amaya Belanger-Quintana, Guillem Pintos-Morell, Beatriz Arconada, Rosa del Campo, José Avendaño-Ortiz

**Affiliations:** 1 Microbiology Department, Hospital Universitario Ramón y Cajal and Instituto Ramón y Cajal de Investigación Sanitaria (IRYCIS), 28034 Madrid, Spain; rql1898@gmail.com (R.B.-H.); anamorenoblanco1993@gmail.com (A.M.-B.); esthepalgo@gmail.com (E.P.); 2 Endocrinology and Nutrition Service, Hospital Universitario Ramón y Cajal, 28034 Madrid, Spain; maria.garriga@salud.madrid.org (M.G.-G.); saioa.vicente@salud.madrid.org (S.V.-S.); 3 CIBER de Enfermedades Infecciosas, Instituto de Salud Carlos III, 28029 Madrid, Spain; 4 Centro de Diagnóstico de Enfermedades Moleculares (CEDEM), Autonomous University of Madrid (UAM), IdiPaz, 28049 Madrid, Spain; pedro.ruiz@inv.uam.es; 5 CIBER de Enfermedades Raras, Instituto de Salud Carlos III, 28049 Madrid, Spain; 6 Unidad de Enfermedades Metabólicas Hospital, CSUR, MetabERN, Pediatric Department, Hospital Universitario Ramón y Cajal, Instituto Ramón y Cajal de Investigación Sanitaria (IRYCIS), 28034 Madrid, Spain; sinziana.stanescu@salud.madrid.org (S.S.); amaya.belanguer@salud.madrid.org (A.B.-Q.); 7 Vall d’Hebron Institut de Recerca (VHIR), Unidad de Enfermedades Raras, Hospital Vall d’Hebron Barcelona Hospital Campus, Comité Médico Consultivo MPS-Lisosomales, 08035 Barcelona, Spain; guillempintos@gmail.com; 8 Federación Española de Enfermedades Raras (FEDER), 28009 Madrid, Spain; b.arconada@enfermedades-raras.org; 9 Faculty of Health Sciences, Alfonso X El Sabio University, Villanueva de la Cañada, 28691 Madrid, Spain

**Keywords:** Sanfilippo syndrome, gut microbiota, *Sus* genes, SCFAs, *Bacteroides thetaiotaomicron*

## Abstract

Sanfilippo syndrome, or mucopolysaccharidosis type III (MPS III), is a rare lysosomal disease caused by congenital enzymatic deficiencies in heparan sulfate (HS) degradation, leading to organ dysfunction. The most severe hallmark of MPS III comprises neurological alterations, although gastrointestinal symptoms (GISs) have also been shown to be relevant in many patients. Here, we explored the contribution of the gut microbiota to MPS III GISs. We analyzed the composition and functionality of the gut microbiota in two MPS III siblings with the same mutation (c.544C > T, c.1080delC, in the SGSH gene) and the same diet, but with differences in their GISs, including recurrent diarrhea in one of them. Using 16S sequencing, we observed that the MPS III patients exhibited decreased alpha diversity and a lower abundance of *Lachnospiraceae* and *Bifidobacteriaceae* accompanied by a higher abundance of the *Ruminococcaceae* and *Rikenellaceae* families than the healthy control subjects. Comparing siblings, we found an increased abundance of *Bacteroidaceae* and a lower abundance of *Ruminococcaceae* and *Akkermansiaceae* in the GIS-free patient. This patient also had a higher relative abundance of *Sus* genes (*SusA*, *SusB*, *SusE*, *and SusG*) involved in glycosaminoglycan metabolism. We found higher HS levels in the stool of the two MPS III patients than in healthy volunteers, particularly in the patient with GISs. Functionally, whole fecal metabolites from the patient with GISs induced oxidative stress in vitro in healthy monocytes. Finally, the *Bacteroides thetaiotaomicron* strain isolated from MPS III stool samples exhibited HS degradation ability. Overall, our results reveal different microbiota compositions and functionalities in MPS III siblings, who exhibited differential gastrointestinal symptomatology. Our study may serve as a gateway to explore the impact of the gut microbiota and its potential to enhance the quality of life in Sanfilippo syndrome patients.

## 1. Introduction

Sanfilippo syndrome, or mucopolysaccharidosis type III (MPS III), is a rare genetic lysosomal disease caused by the absence of or deficiency in one of the four enzymes responsible for breaking down heparan sulfate (HS) [1]. HS accumulation in the lysosomes triggers progressive damage in various tissues, especially in the central nervous system, leading to neurological impairments [2]. Evidence indicates that MPS III alterations are not only restricted to the lysosomes but also affect other organelles and extracellular compartments, causing neuroinflammation and multiorgan symptomatology [3,4,5]. These secondary effects could explain the results of the treatments tested for this disease, including enzyme replacement therapy and substrate reduction, which were not successful in alleviating symptoms, despite reducing HS levels [6,7,8].

The main causes of death in MPS III patients are severe respiratory infections (pneumonia with secondary respiratory failure) in almost half of all cases, followed by cardiorespiratory failure and gastrointestinal complications [9]. Regarding the latter, a recent systematic review pointed out that gastrointestinal symptoms (GISs) may be underrecognized in MPS III, despite evidence revealing their impact on clinical outcomes [10]. Indeed, mouse models exhibit intestinal alterations including greater duodenum length and weight, as well as increased submucosal thickness in the MPS III intestine compared to the wild type [11]. Nevertheless, the impact of GISs and gut-related factors on MPS III pathophysiology remains unexplored.

The term microbiota is used to define the set of microorganisms (mainly bacteria and fungi) that coexist in different tissues of our body [12]. The most prominent and studied is the gut microbiota, which is known to play a key role in metabolism [13]. Microbiota alterations have been found not only in diseases directly related to the gastrointestinal system but also in chronic inflammatory diseases and even neurological and neurocognitive disorders such as autism [14,15]. In the context of mucopolysaccharides metabolism, it has been found that commensal bacteria, especially *Bacteroides thetaiotaomicron*, have an HS degradation system comprising heparinase, lyase, and sulfatases under the known polysaccharide utilization loci (PUL) for the starch utilization system (Sus) genes [16,17,18,19]. Despite increasing knowledge in other diseases, there is currently no information on the composition and distribution of the gut microbiota in patients with MPS III.

The role of the microbiota in health and disease also involves the modulation of inflammation and immune system responses. It is well known that microbiota-derived compounds like short-chain fatty acids (SCFAs) can exert systemic functions through immunomodulation [12,20]. Studies in mucopolysaccharidoses animal models have proposed that the activation of the innate immune cells through HS in MPS is an underlying cause of neuroinflammation and oxidative stress [21,22,23,24]. The role of microbial-derived metabolites in the hallmarks of MPS III has not yet been studied.

Herein, we study the composition and functionality of the gut microbiota of two siblings with Sanfilippo syndrome caused by the same mutation in the *N-sulfoglucosamine sulfohydrolase* (SGSH) gene but exhibiting differential GISs. We analyze the presence of PUL genes, as well as the levels of HS, and SCFAs in the feces. Fecal metabolites exerted different effects on immune system cells in vitro. In addition, we isolated *Bacteroides* spp. strains to test their ability to metabolize HS. Overall, our study highlights the role of microbiota in the GISs of Sanfilippo syndrome and opens a window for evaluating targeted probiotics to minimize GISs in these patients.

## 2. Results

### 2.1. Patient Description: Clinical Similarities and Differences between the Two MPS-IIIA Siblings

Two siblings harboring p.R182C (c.544C > T) and V361Sfs*52 (c.1080delC) in the SGSH gene, causing severe MPS III type A, were included in this study. Both siblings shared some clinical characteristics, including a severe intellectual disability, a behavioral disorder, epilepsy, a sleep disorder, thickened atrioventricular and sigmoid valves, and mild aortic regurgitation (Table 1). Blood tests showed normal cell counts and chemistry analytes, except for slightly increased alanine aminotransferase (ALT) and gamma-glutamyl transferase (GGT) values (Table 1). Both patients followed the same diet of pureed foods, with no gluten or lactose restrictions (Table 1).

Sibling 1 is a 13-year-old girl diagnosed at the age of 2 years after hepatomegaly, macrocephaly, and high levels of glycosaminoglycans (GAGs) in the urine. Her current treatment options include carbamazepine, trihexyphenidyl, melatonin, clonazepam, vitamin D, and tizanidine. She showed severe motor problems characterized by an inability to walk and stand upright, with a total Meyer functional scale [25] of 0–1 (Table 1).

Sibling 2 is a 12-year-old boy diagnosed at the age of 2 years after his older sister was diagnosed. He was evaluated for hepatomegaly accompanied by elevated liver enzyme levels. He presented with better motor skills than his sister. However, he has exhibited recurrent diarrhea, limiting his quality of life. His current treatment is the same as that of his sister, except for the last two years, when he started levomepromazine treatment as a neuroleptic drug.

### 2.2. Microbial Composition, PUL Genes Abundance in MPS III Patients

**Figure 1:**
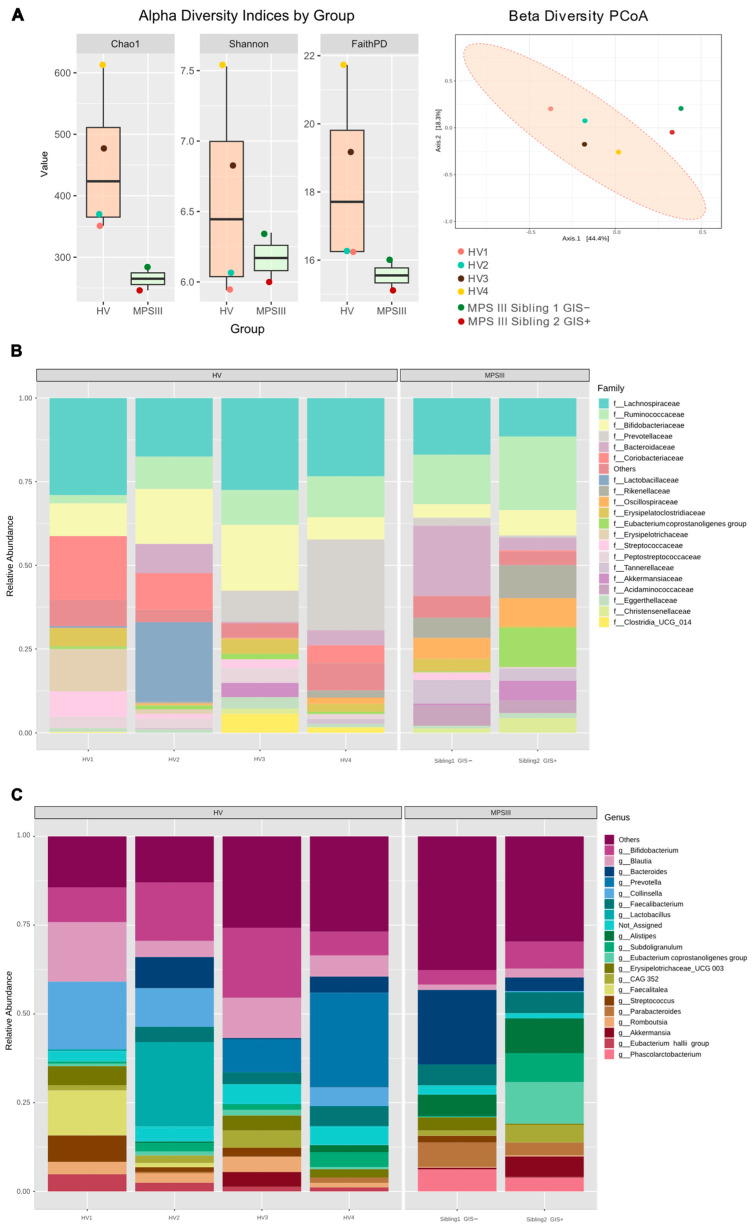
Analysis of gut microbiota in healthy volunteers (HVs) and patients with Sanfilippo syndrome (MPS III). (**A**) The alpha diversity, Chao1, Shannon, and Faith PD indexes and the principal coordinates analysis (PCoA) plot of Bray−Curtis beta diversity are shown. Microbial composition was analyzed by 16S rRNA amplicon sequencing of feces from 4 HVs and the MPS III siblings included in the study. Relative abundance of families (**B**) and the 20 most abundant genera (**C**). f__, family; GIS, gastrointestinal symptoms; g__, genus.

As mentioned above, despite the two siblings harboring the same mutation and consuming a similar diet, one of the major differences was that while patient 2 had frequent diarrhea, sibling 1 had no GISs. Thus, we compared gut microbiota composition between the two MPS III siblings and four age-matched healthy volunteers (HVs) carrying a restriction-free diet. 16S gene sequencing of fecal samples revealed lower diversity in the MPS III patients based on Chao1 and FaithPD indexes (Figure 1A). Similarly, beta diversity analysis using the Bray–Curtis distance revealed the MPS III microbiota composition separated from the HV cluster (Figure 1A). In terms of abundance, we found lower *Lachnospiraceae* and *Bifidobacteriaceae* abundance, along with a higher abundance of the *Ruminococcaceae* and *Rikenellaceae* families in MPS III individuals (Figure 1B). These changes corresponded with a lower abundance of *Bifidobacterium*, *Blautia*, and *Collinsella* and a higher abundance of *Parabacteroides* and *Alistipes* (Figure 1C). In the comparison of the siblings, regardless of the diversity indexes analyzed, alpha diversity was similar, although it was always higher in the GIS-free sibling (Chao1 284 vs. 246; Faith-PD 15.99 vs. 15.11; Shannon 6.35 vs. 5.99, Figure 1A). An increased abundance of *Bacteroides* was found in this patient (18.4% vs. 3.5%), with the *Bacteroidaceae* family and *Bacteroides* genus being dominant (Figure 1). In contrast, the *Ruminococcaceae* family was the most abundant in the patient with GISs (21.0% vs. 13.0%). Differences in *Akkermansia* and *Eubacterium coprostanoligenes* also stood out, with both being more abundant in the sibling with GISs.

**Figure 2:**
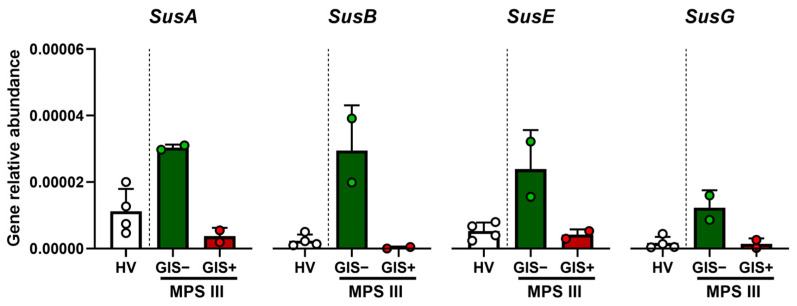
Relative abundance of PUL (*SusA*, *SusB*, *SusE*, *and SusG*) genes assessed by qPCR from stool samples of MPS III siblings and healthy volunteers. GIS, gastrointestinal symptoms; HV, healthy volunteers; MPS III, Sanfilippo syndrome siblings. Each dot represents the average of 3 technical replicates and bars are expressed as mean ± SD. HV, *n* = 4 stool samples from 4 different healthy volunteers; GIS+, *n* = 2 two different day samples from the GIS+ MPS III patient.; GIS−, *n* = 2 two different day stool samples from the GIS+ MPS III patient.

Since the abundance of *Bacteroides* genus was the clearest difference between siblings and *Bacteroides* spp. may possess PUL, we decided to analyze the relative abundance of specific PUL-related genes in patients’ feces. The GIS-free sibling, who showed higher *Bacteroides* spp. abundance, also exhibited a higher relative abundance of the *SusA*, *SusB*, *SusE*, and *SusG* genes (Figure 2). Globally, our data revealed changes in microbiota composition between the siblings, mainly summarized by lower *Bacteroides* spp. levels in the GIS+ patient.

Given that one of the differences between MPS III patients was the administration of levomepromazine, we decided to explore the antimicrobial activity of this drug on *Bacteroides* spp. and other commensal strains isolated from the microbiota of the patients and healthy volunteers. We found that the minimum inhibitory concentration (MIC) was 156 µg/mL for *Escherichia coli* and *Enterococcus faecium* strains, and was slightly lower for *Bacteroides fragilis* and *Bacteroides thetaiotaomicron* strains, at 78 and 39 µg/mL, respectively. Thus, *Bacteroides*, especially *B. thetaiotaomicron*, appears to be more susceptible to levomepromazine, which is precisely the treatment received by the patient with a lower abundance of *Bacteroides* spp. compared to his sibling.

### 2.3. Microbial-Derived Metabolites and HS Levels in Stool: Role in Oxidative Stress

**Figure 3:**
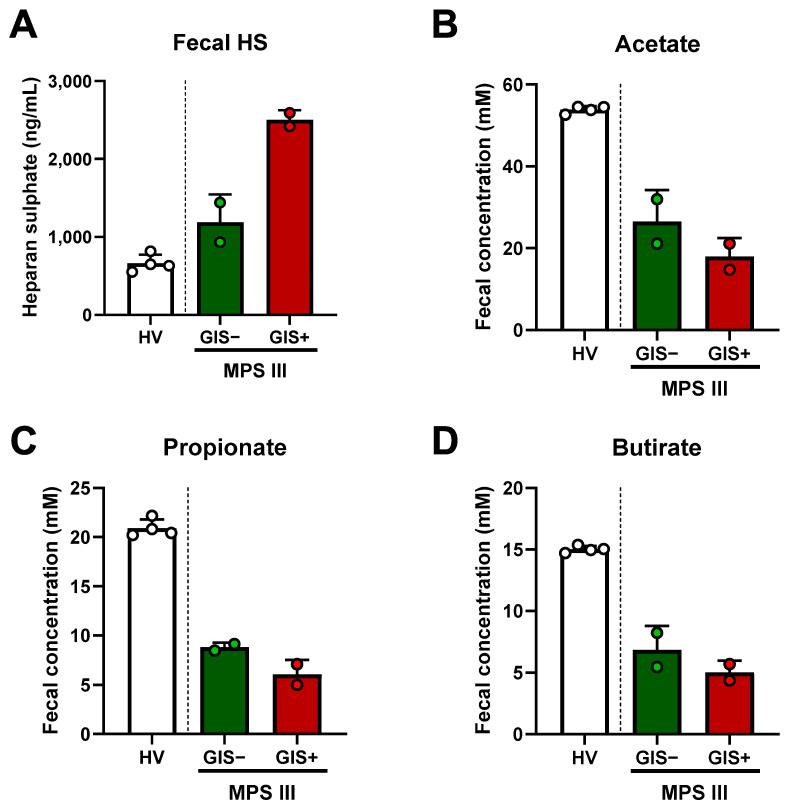
Fecal levels of heparan sulfate (HS) (**A**), acetate (**B**), propionate (**C**), and butyrate (**D**) were determined by ELISA and gas chromatography coupled with mass spectrometry (GC-MS), respectively. Each dot represents the average of 3 technical replicates and bars are expressed as mean ± SD. HV, *n* = 4 stool samples from 4 different healthy volunteers; GIS+, *n* = 2 two different day samples from the GIS+ MPS III patient.; GIS−, *n* = 2 two different day stool samples from the GIS+ MPS III patient. GIS, gastrointestinal symptoms; HV, healthy volunteers; MPS III, Sanfilippo syndrome siblings.

The determination of total GAGs and HS in the urine is a widely used tool for MPS III diagnosis. However, fecal levels have not yet been studied. As expected, patients with MPS III showed higher fecal HS levels than the healthy volunteers. Interestingly, the GIS+ sibling exhibited higher levels than the GIS-free patient (Figure 3A). In contrast, the total GAG levels in urine were lower in the GIS+ patient, corresponding with the higher sulfamidase activity in his leukocytes than in his sister (Table 1). We also determined the SCFA concentration in feces as a functional marker of the microbiota, resulting in lower concentrations of acetate, propionate, and butyrate in MPS III, especially in the GIS+ sibling (Figure 3B–D).

**Figure 4:**
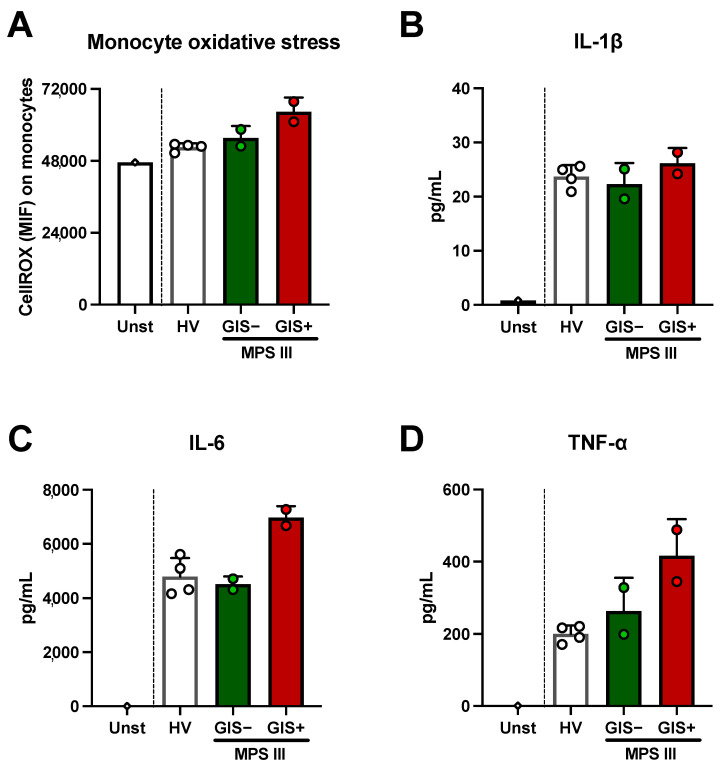
Effects of MPS III fecal metabolites on healthy monocytes. Monocytes from 5 healthy volunteers’ peripheral blood were isolated and stimulated for 24 h with fecal metabolites from patients with MPS III. (**A**) Oxidative stress in gated monocytes established by Green CellROX staining was analyzed by means of flow cytometry. Levels of IL-1β (**B**), IL-6 (**C**), and TNF-α (**D**) in monocyte culture supernatant are shown. Each dot represents the average from experiments with 5 independent human healthy monocytes and bars are expressed as mean ± SD. HV, *n* = 4 stool samples from 4 different healthy volunteers; GIS+, *n* = 2 two different day stool samples from the GIS+ MPS III patient.; GIS−, *n* = 2 two different day samples from the GIS+ MPS III patient. Unst, unstimulated.

Once we had observed that the fecal levels of HS and SCFAs from the MPS III patients showed anomalies compared to the HVs, we moved on to studied whether fecal HS and SCFAs could have effects on innate immune cells. To this end, we stimulated monocytes from healthy volunteers with MPS III fecal metabolites. We found that metabolites from the GIS+ patient induced higher oxidative stress than those from the GIS− patient, which were closer to the values obtained from the HVs (Figure 4A). Regarding inflammation, we found IL-1β production after fecal metabolite stimulation was similar between patients and HVs (Figure 4B). However, IL-6 and TNF-α production was higher in the supernatant from monocytes stimulated with fecal metabolites from the GIS+ patient than in both the GIS− patient and the HVs (Figure 4C,D).

### 2.4. Bacteriodes thetaiotaomicron Isolate Exhibited In Vitro HS Degradation Ability

**Figure 5:**
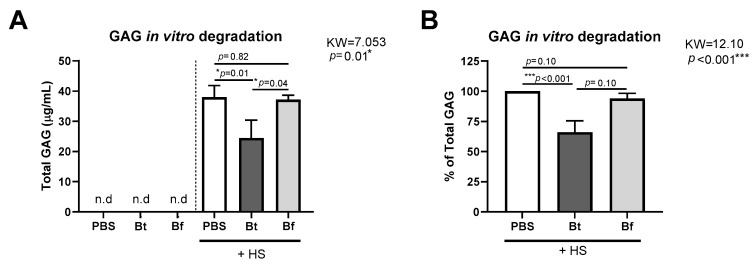
HS degradation activity of bacteria isolated from the stool of MPS III patients. *Bacteroides* spp. strains were isolated from stool samples of the two MPS III siblings included in this study. Bacteria were cultured in the presence of 75 µg/mL of HS at 37 °C under anaerobic conditions for 48 h. (**A**) Total GAG in the culture supernatant determined using the 1,9-dimethylmethylene blue procedure is shown. (**B**) Percentage of total GAG normalized to the PBS control. Bf, *Bacteroides fragilis*; Bt, *Bacteroides thetaiotaomicron*; n.d., not detected. KW, Kruskal–Wallis statistic; * *p*-value < 0.05 and *** *p*-value < 0.001 analyzed using the Kruskal–Wallis ANOVA test followed by Dunn’s multiple comparisons test. Data are expressed as mean ± SD from *n* = 4 independent experiments.

Once we had observed that the GIS+ sibling exhibited lower *Bacteroides* spp. and higher HS in their feces, we analyzed whether some of the commensal bacteria of the MPS III patients could degrade HS. To this end, we decided to isolate *Bacteroides* spp. from the patients’ stool. We isolated *Bacteroides fragilis* and *Bacteroides thetaiotaomicron* strains. We found that the *B. thetaiotaomicron* strain was able to degrade HS in vitro, as the GAG levels in the culture supernatant were decreased compared with the PBS control (Figure 5). In contrast, *B. fragilis* did not exhibit this ability as the levels were similar to those of the control (Figure 5). Accordingly, the capacity of *B. thetaiotaomicron* to degrade HS could align with the observation that the patient with the highest quantity of *Bacteroides* spp. also exhibited lower levels of HS in their stool.

## 3. Discussion

Sanfilippo syndrome, or MPS III, is a rare disorder that primarily affects the metabolism of HS, causing organ malfunctions and ultimately resulting in premature death [9]. There are no curative therapies available for MPS III, and current patient management strategies only address the symptoms [26]. Research on this pathology has traditionally focused on the severe neurological manifestations; however, recent evidence indicates other features, such as the gastrointestinal alterations that are underestimated in MPS III, despite being a relevant cause of disability and morbidity [10]. The gut microbiota may play a role in gastrointestinal symptomatology; however, this remains totally unexplored.

Alterations in the microbiota composition have been associated with several diseases, including neurological disorders such as autism, Parkinson’s disease, and multiple sclerosis [27,28,29,30]. Here, we found an overall decrease in alpha diversity indices and a lower abundance of *Lachnospiraceae* and *Bifidobacteriaceae*, along with *Bifidobacterium*, *Blautia*, and *Colinsella* genera, in the microbiota of patients with MPS III compared to healthy controls. In contrast, a higher abundance of the *Ruminococcaceae* and *Rikenellaceae* families was found. Alpha diversity, a measure of microbial community complexity, is crucial for maintaining gut homeostasis and overall health [31,32,33]. Regarding composition, a decrease in *Bifidobacterium* abundance during the initial colonization of the infant gut can hinder the normal development of the gut microbial community and potentially have a negative impact on the host’s health [34]. *Blautia*, *Lachnospiraceae*, and *Ruminococcaceae* are associated with intestinal barrier integrity and anti-inflammatory properties through SCFA production [12]. A possible balance between *Lachnospiraceae* and *Ruminococcacea* could occur in MPS III, as the former is decreased and the latter is increased in patients.

In our study, we compared the microbiota composition of two MPS III siblings with the same mutation and diet who exhibited differing gastrointestinal manifestations and differential microbiota; a slightly lower alpha diversity, a lower *Bacteroides* abundance, and a higher abundance of the *Akkermansiaceae* and *Ruminococcaceae* families in the patient with GISs. Some of these bacteria play different roles in gut function and metabolism. Among them, the most interesting is *Bacteroides* spp., as they are metabolically active symbionts by their PULs [17,35,36,37]. The PUL from *Bacteroides* spp. bacteria include *Sus* genes that govern glycan uptake and specifically target HS, chondroitin sulfate, dermatan sulfate and hyaluronic acid [37,38]. *SusA*, *SusB*, and *SusG* codify for a pullulanase GH13 α1,4-glucosidase, a GH97 α1,6-glucosidase and a cell-membrane α-amylase, respectively, which are sufficient to hydrolyze all of the linkages in glycosamianoglycan polysaccharides [35,39,40]. *SusE* is part of the surface receptor complex that facilitates starch uptake independent of starch binding [36,41,42]. Despite the fact that these kinds of genes could be involved in the glycosaminoglycans metabolism, including HS, their relationship with the gastrointestinal discomfort in MPS III patients has not been previously considered. Here, we found a lower abundance of *Bacteroides* spp. and *SusA*, *SusB*, *SusE*, and *SusG* genes in the stool of the MPS III patient with recurrent diarrhea, suggesting a potential relationship between microbial *Sus* genes and GISs. It is noteworthy that the increase in *Bacteroides* and *Sus* genes in the child without GISs would be above the values of the healthy controls. An increase in this genus favored by the high amount of HS in the patient gut environment could not be ruled out.

The molecular basis of MPS III is the accumulation of HS in lysosomes; however, their accumulation in extracellular compartments and organ lumens seems to be one of the causes of multiorgan symptomatology [3,4,5]. Indeed, the determination of glycosaminoglycans in urine is one of the primary diagnostic tools in MPS III and other mucopolysaccharidoses [43]. Beyond urine, HS is shown to be elevated in the plasma and cerebrospinal fluid of Sanfilippo patients [44]. However, information on more complex matrices such as feces is not available. Our data showed that the levels of HS in stool samples from the two patients with MPS III included in this study are higher than those from healthy individuals. Furthermore, it is noteworthy that the sibling with gastrointestinal symptoms and lower *Bacteroides* spp. had the highest fecal levels of HS, despite having lower urine levels and higher sulfamidase activity. As both patients followed the same diet, we speculate that the differences in fecal levels reflect microbiota-derived HS metabolism. In this sense, it is well known that *Bacteroides’* ability to target highly sulfated host glycans is crucial to their success in the gut environment [37,45]. Given the ability of *Bacteroides* spp. to metabolize glycosaminoglycans [16,17,18,19], the possible relationship between their abundance and fecal HS levels should be further explored.

In recent years, there has been growing interest in investigating not only the microbiota’s composition but also its functionality. One of the main functions of the gut microbiota is the digestion of fiber and resistant starch and their transformation into SCFAs [46]. These fatty acids are known to regulate inflammation and mitochondrial function, reducing oxidative stress [46,47,48]. Here, we found that SCFAs were lower in the two MPS III included in this study than in the healthy volunteers and even lower in the patient with recurrent diarrhea. Although the differences between the GIS+ and GIS− patients are minor, there is a possibility that they are the consequence of changes in the abundance of *Lachnospiraceae*, *Blautia*, and *Bifidobacteriaceae* mentioned above [12]. Functionally, our in vitro data suggest that the fecal metabolites of the GIS+ individual induced more oxidative stress along with a higher inflammatory response in healthy monocytes than the metabolites from all other individuals included in this study. In this line, the excess level of HS has been linked to inflammasome activation, impaired autophagy, and an increase in mitochondrial dysfunction and ROS production in the innate immune cells of MPS III patients [23,24].

Overall, our findings reveal disparities in microbiota composition and function according to gastrointestinal symptoms. Thus, it might be worthwhile to explore the potential benefits of dietary influence or probiotic use to enhance the quality of life of Sanfilippo syndrome patients. A clinical case study reported that a diet with long-chain fatty acid restrictions and rich in medium-chain triglycerides caused sustained clinical improvement in a Sanfilippo syndrome type B patient with chronic diarrhea [49]. Nonetheless, currently, there are no clinical guidelines or consensus on nutritional recommendations or probiotic supplementation in MPS III. Here, we isolated a *B. thetaiotaomicron* strain from the microbiota of patients with MPS III, which showed good capacity to degrade HS in vitro. Notably, *B. thetaiotaomicron* has been found to prevent neuroinflammation and cognitive decline through its role in carbohydrate foraging activities [50]. Studies also indicate that *B. thetaiotaomicron*, along with *Akkermansia muciniphila*, can regulate the intestinal epithelial barrier, unveiling its potential as a new generation of probiotics [51]. An autologous probiotic strategy based on the isolation of relevant patient strains to enrich specific bacterial species may reduce the risk of unexpected toxigenic effects.

The major limitation of our study is that it included only two patients with MPS III, making it difficult to generalize the results to all MPS III patients. As it is a rare disease, it is difficult to perform studies with large cohorts. This is particularly challenging because there are four different types of MPS III depending on the gene affected, each one with a vast number of possible mutations corresponding to enzymatic activities [52]. It is even more complicated to study microbiota composition, as diet or feeding type may be confounding variables. The main strength of our study is that the patients were siblings with the same mutation and diet, thus minimizing differences in their genetic background and external factors. Nonetheless, the treatment could have an impact on the microbiota. Here, we found that levomepromazine, the unique treatment difference between the siblings, has inhibitory effects on gut microbiota strains with slightly higher activity in *Bacteroides* spp. (MIC 39–78 µg/mL). Previous research revealed that the MICs of levomepromazine and other phenothiazines are around 32–64 μg/mL for *S. aureus*, and are slightly higher (from 64 to 128 μg/mL) for *E. coli*, and *K. pneumoniae* [53]. The influence of sex on the gut microbiota cannot be discarded either. Studies have shown that sex shapes the abundance of taxa in the gut microbiota, with females having a slightly higher abundance of *Bacteroides* compared to males, attributed to the hormonal regulation of microbe-controlling mechanisms [54,55]. Our study indicated a different trend, as *Bacteroides* was found to be increased in the female patient without gastrointestinal symptoms.

In conclusion, our results suggest, for the first time, a role of the gut microbiota in the gastrointestinal symptoms of MPS III. Specifically, *Bacteroides* spp., through their PUL and *Sus* genes involved in the uptake and metabolism of extracellular HS, emerge as an interesting genus to be considered in MPS III patients with gastrointestinal symptoms. Further research into the specific mechanisms and the impact of the gut microbiota on other MPS III symptoms, such as neurocognition, is warranted to fully understand their potential role.

## 4. Materials and Methods

### 4.1. Participants and Study Design

Two siblings diagnosed with MPS III and four age- and sex-matched HVs were included in this study. From each patient, two stool samples from two different days were collected. Once in the laboratory, the feces samples were divided into aliquots and immediately frozen at −80 °C. Aliquots were used to determine the whole bacterial composition by means of 16S rDNA sequencing, in order to isolate viable potential HS-consuming bacteria/*Bacteroides* spp. via conventional microbiological culture, to determine SCFA and HS, and to study the induction of oxidative stress in monocytes. This study was conducted in accordance with the ethical guidelines of the 1975 Declaration of Helsinki and was approved by the Hospital Universitario Ramón y Cajal Clinical Research Ethics Committee. Informed consent was obtained from all legal guardians of the patients and the HVs.

### 4.2. Microbiota Composition by 16S rDNA Sequencing

An aliquot of each stool sample (0.5 g) was solubilized in sterile water (5 mL). DNA was extracted using the Speedtool Tissue DNA Extraction Kit (Biotools, San Francisco, CA, USA). Amplicons including the V3 and V4 regions of the 16S rDNA gene were sequenced on a MiSeq platform (Illumina, San Diego, CA, USA) with a read length of 2 × 300 bp. We followed the 16S Metagenomic Sequencing Library Preparation protocol (Illumina; Cod. 15,044,223 Rev. A). Sequence quality control was undertaken using DADA2 [56], and taxonomic assignment was performed using QIIME2 (amplicon distribution version 2023.9, https://docs.qiime2.org/2023.9/ accessed on 15 December 2023, [57]) and the SILVA 138 database [58]. Alpha and beta diversity were assessed using the q2-diversity add-on of QIIME2, which was performed after normalizing the samples through rarefaction (subsampling without replacement). 16S rDNA sequencing data were archived in GenBank (BioProject PRJNA1113948), while the amplicon sequence variant (ASV) data are presented in Supplementary Table S1.

### 4.3. Relative Abundance of Polysaccharide Utilization Loci by qPCR

The relative abundances of *SusA*, *SusB*, *SusE*, *SusG*, and 16S rRNA (total bacterial abundance) were determined using quantitative PCR (qPCR). The primer pairs used to amplify these genes are listed in Supplementary Table S2. DNA from stool samples was extracted as described above. qPCR was performed using PowerUp™ SYBR™ Green Master Mix (Applied Biosystems, Waltham, MA, USA) with a total volume of 20 µL, a total primer concentration of 500 nM, and a total stool DNA concentration of 1 ng/µL. The reaction was run in duplicate in a QuantStudio™ 6 Flex Real-Time PCR System (Applied Biosystems) on MicroAmp™ Optical 96-Well plates (Applied Biosystems). The detection limit was set at a threshold cycle (Ct) lower than 35. If the Ct value exceeded 35, the gene abundance was regarded as 0. The relative abundance of each *Sus* gene was calculated using the ΔCt method [59,60]. Data were normalized by subtracting the 16S rDNA Ct value from the Ct values for the *Sus* genes in each sample to calculate ΔCt values, which were expressed as 2 ^[Ct (16S rRNA qPCR) − Ct (target gene qPCR)]^.

### 4.4. Levomepromazine MIC Determination

The MIC of levomepromazine (Sanofi, Paris, France) was determined by means of the microdilution method in 96-well U-bottom plates (Corning, New York, NY, USA). The bacterial strains tested included *E. faecium*, *E. coli*, *B. fragilis*, and *B. thetaiotaomicron* strains isolated from the stool of patients and healthy volunteers (Supplementary Table S3). Bacteria were identified using MALDI-TOF (Biotyper^®^, Bruker, Bremen, Germany), and *B. thetaiotaomicron* were confirmed by means of PCR using *B. thetaiotaomicron* species-specific BTH primers described by Teng et al. [61]. Briefly, a 0.5 McFarland scale inoculum was prepared in a sterile saline solution for each strain. This initial suspension was 1:100 diluted in Mueller–Hinton broth (Millipore, Burlington, MA, USA). The levomepromazine concentrations tested ranged from 2 to 2500 µg/mL via serial dilution. After an overnight incubation at 37 °C, the wells were examined for visible bacterial growth. The lowest concentration of levomepromazine that prevented bacterial growth was defined as the MIC.

### 4.5. Fecal HS and SCFAs Determination

For fecal HS determination, 100 mg of feces was dissolved in 10 mL of PBS and centrifuged for 15 min at 2145× *g*. The supernatants were utilized for HS determination using the Human HS ELISA Kit EH4010 (FineTest, Wuhan, China) following the manufacturer’s protocol. SCFAs were quantified by gas chromatography coupled with mass spectrometry (GC-MS Trace 1300, Thermofisher, Dreieich, Germany) with a TG-WAXMS GC column (Thermofishe, Dreieich, Germany) as previously described [62]. Briefly, 30 mg of feces was extracted using 10 volumes of ethanol and treated with 0.8 M NaOH freshly prepared to perform alkaline vacuum concentration (SpeedVac™ SPD121P, Thermo Fisher Scientific, Dreieich, Germany). Immediately before injection, samples were acidified with 0.6 M succinic acid at a ratio of 1:6 in ethanol to allow volatility. Analytes were detected by means of selected ion monitoring (SIM) and using D7 deuterated butyric acid as an internal standard.

### 4.6. Monocyte Cell Culture and Fecal Metabolite Oxidative Stress and Inflammatory Assay

Peripheral blood mononuclear cells (PBMCs) from the blood of healthy volunteers were isolated using a Ficoll-Plus gradient (GE Healthcare Bio-Sciences, Chicago, IL, USA). The human monocyte population was enriched from PBMCs by means of adherence selection in a serum-free RPMI medium (Gibco, Waltham, MA, USA) as previously described [63,64]. After that, adherent monocytes were washed twice with PBS and cultured in RPMI supplemented with 10% fetal bovine serum (FBS). To obtain fecal metabolites, 0.5 g of feces from MPS III patients was solved in 10 mL of saline, centrifuged at 5000× *g* for 15 min, and the supernatant was filtered twice with a 0.22 µm polyethersulfone membrane filter (Jet BIOFIL) to discard remnant bacteria. The supernatants were used to stimulate monocyte cultures at a 1:100 (*v*/*v*) dilution in complete RPMI for 24 h. For oxidative stress determination, cells were labeled with CellROX™ Green Reagent (Invitrogen, Eugene, OR, USA) and analyzed via flow cytometry using CytoFLEX (Beckman Coulter, Brea, CA, USA). Cytokine levels in monocyte culture supernatant were measured by means of flow cytometry using the LEGENDplex™ HU Essential Immune Response Panel (BioLegend, San Diego, CA, USA) in a BD FACSCanto II (BD Biosciences, San Jose, CA, USA) and analyzed using the LEGENDplex Data Analysis Software Suite v2023-02-15 (Qognit, Inc., San Carlos, CA, USA).

### 4.7. Bacteroides Spp. Isolation and In Vitro HS Degradation Assay

For *Bacteroides* spp. isolation, feces were diluted in saline and streaked on *Bacteroides* bile esculin (BBE, from HiMedia) agar plates supplemented with gentamicin, specifically recommended for *Bacteroides* selective isolation [65]. Afterwards, colonies were identified using matrix-assisted laser desorption ionization-time of flight mass spectrometry (MALDI-TOF, Bruker-Daltonics, Germany) and via PCR using *Bacteroides thetaiotaomicron* species-specific BTH primers [61]. A *B. fragilis* strain isolated from sibling 1 and a *B. thetaiotaomicron* strain isolated from sibling 2 were used for the in vitro HS degradation assay. From a fresh 24 h agar plate, bacteria were adjusted to 1 McFarland in PBS, seeded in a 96-well clear round-bottomed flask (Corning) and treated with 75 μg/mL of HS from bovine kidney (Sigma, Burlington, MA, USA). Then, bacteria were cultured at 37 °C under anaerobic conditions for 48 h. The total sulphated glycosaminoglycan level in the culture was measured via spectrophotometry using the 1,9-dimethylmethylene blue assay [66].

### 4.8. Statistical Analysis

For HV vs. MPS III comparisons, the Mann–Whitney test was used. For multiple-group comparisons in the stimulation experiments, the Kruskal–Wallis ANOVA test followed by Dunn’s ad hoc multiple comparisons test was used. All statistical analyses were performed using the GraphPad Prism 8 software (San Diego, CA, USA).

## 5. Conclusions

Gut microbiota composition and function showed differences between the two siblings with different gastrointestinal manifestations. In summary, the sibling with chronic diarrhea exhibits lower abundance of *Bacteroides* spp. and *Sus* genes along with higher fecal HS and lower SCFAs than the symptom-free sibling. The in vitro findings including oxidative stress induction on innate immune cells and *Bacteroides* spp. strain isolation suggest the potential implications of these alterations. Overall, our data open a window in the study of the role of the gut microbiota in ameliorating clinical manifestations and improving the quality of life of patients with Sanfilippo syndrome.

## Figures and Tables

**Table 1:** Summary description of patients with MPS III included in this study.

	Sibling 1	Sibling 2
Age (years)	13	12
Sex	Female	Male
Age at diagnosis (years)	2	2
Symptoms	Intellectual disability, behavioral disorder, epilepsy, sleep disorder, loss of walking and standing ability	Intellectual disability, behavioral disorder, epilepsy, sleep disorder, and recurrent diarrhea
Current treatment	Carbamazepine, tizanidine, trihexyphenidyl, vitamin D, clonazepam, melatonin	Carbamazepine, tizanidine, vitamin D, levomepromazine, clonazepam, melatonin
Urine total GAGs (mg/mmol creatinine)	20.25	11.98
Sulfamidase activity (nmol/mL/17 h)	0.15	0.38
ALT (U/L)	42	50
GGT (U/L)	39	44
Meyer scale	0–1	
- Motor	0	1–2
- Speech	0	0
- Cognitive	0–1	1
Daily diet		
- Energy (Kcal)	1080	1092
- Fat (g per day)	33	33
- Proteins (g)	43	43
- Carbohydrates (g)	147	149
- Fiber (g)	11.50	11.50
Calcium (mg)	632	632

ALT, alanine transaminase; GAGs, glycosaminoglycans; GGT, gamma-glutamyl transpeptidase.

## Data Availability

The 16S rDNA sequencing data were archived in GenBank (BioProject PRJNA1113948). The raw data supporting the conclusions of this study will be made available by the authors upon reasonable request.

## Supplementary Materials

The following supporting information can be downloaded at: https://www.mdpi.com/article/10.3390/ijms25168856/s1.
