# Supplementary material for: The Role of the Gut Microbiota in Sanfilippo Syndrome’s Physiopathology: An Approach in Two Affected Siblings
Source: Int J Mol Sci. 2024 Aug 14;25(16):8856. doi: 10.3390/ijms25168856 (PMC11354487; doi:10.3390/ijms25168856)
Supplement: Supplementary file 1 [file ijms-25-08856-s001.zip › ijms-3095115-supplementary/Supplementary Tables S2 and S3.pdf]

Supplementary Materials

The role of gut-microbiota in Sanfilippo Syndrome physiopathology: an approach in two affected siblings

Raquel Barbero-Herranz, María Garriga-García, Ana Moreno-Blanco, Esther Palacios, Pedro Ruiz-Sala, Saioa Vicente-Santamaría, Sinziana Stanescu, Amaya Belanger-Quintana, Guillem Pintos-Morell, Beatriz Arconada, Rosa del Campo, José Avendaño-Ortiz

Supplementary Table S2. Sequences of primers used in this study.

Gene	Forward	Reverse
16S rDNA	AGGATTAGATACCCTGGTAGTCCA	AGGCCCGGGAACGTATTCAC
Sus A	CGTAATGAGCCTTTCGCACG	TTCCTGAATCGGGTTGAGCC
Sus B	TTCCGCTTCTGTACGCAACT	TCATATTCTGTACCGCGGGC
Sus E	AGTGTTGGAAAGCACCTCCC	GTAGGGAGTTCTGCCTGCTC
Sus G	CAAACGGGCAGACATCTCCT	TACAGGGGTGGATGGGAGAG
BTH	TGGAGTTTTACTTTGAATGGAC	CTGCCCTTTTACAATGGG

Supplementary Table S3. Bacterial strains isolated and used in this study.

Strain	Specie	Origin
1	*Bacteroides fragilis*	MPS III sibling 1 stool
2	*Bacteroides thetaiotaomicron*	MPS III sibling 2 stool
3	*Escherichia coli*	Healthy volunteer 1 stool
4	*Escherichia coli*	MPS III sibling 2 stool
5	*Enterococcus faecium*	Healthy volunteer 1 stool
